# Supplementary material for: Tractor workflow: a scalable Nextflow framework for local ancestry-aware genome-wide association studies
Source: Bioinformatics. 2026 Mar 16;42(5):btag124. doi: 10.1093/bioinformatics/btag124 (PMC13197121; doi:10.1093/bioinformatics/btag124)
Supplement: btag124_Supplementary_Data [file btag124_supplementary_data.zip › Supplement_Data/SupplementaryTablesAndFigures.pdf]

Supplementary Tables and Figures

	LiftOver						LiftOver (Post-Filters)		SHAPEIT5 Phasing (filter)	
Chrom	Original number of variants	Variants lifted over	Percent Variants lifted over	Variants failed to lift over	Percent Variants failed to lift over	Variants lifted over, but with mismatched ref alleles	LiftOver Filtered Variants	Percent LiftOver Filtered Variants	Variants after SHAPEIT5 phasing (MAF filter: 0.5%)	Percent Variants after SHAPEIT5 phasing (MAF filter: 0.5%)
chr1	1,135,111	1,127,990	99.37%	4,399	0.63%	2,722	1,126,431	99.24%	1,122,580	98.90%
chr2	1,208,210	1,206,077	99.82%	1,605	0.18%	528	1,205,894	99.81%	1,202,383	99.52%
chr3	1,032,523	1,029,864	99.74%	2,088	0.26%	571	1,029,735	99.73%	1,027,060	99.47%
chr4	1,051,797	1,050,900	99.91%	507	0.09%	390	1,050,880	99.91%	1,047,568	99.60%
chr5	941,101	940,682	99.96%	337	0.04%	82	940,662	99.95%	938,568	99.73%
chr6	941,665	934,115	99.20%	6,402	0.80%	1,148	933,839	99.17%	932,035	98.98%
chr7	855,823	848,016	99.09%	6,079	0.91%	1,728	847,734	99.05%	846,046	98.86%
chr8	805,762	804,219	99.81%	447	0.19%	1,096	804,200	99.81%	802,237	99.56%
chr9	639,850	636,333	99.45%	3,068	0.55%	449	635,364	99.30%	633,797	99.05%
chr10	733,631	725,522	98.89%	7,744	1.11%	365	725,174	98.85%	722,919	98.54%
chr11	724,378	722,955	99.80%	981	0.20%	442	722,804	99.78%	720,049	99.40%
chr12	696,024	692,817	99.54%	3,070	0.46%	137	692,759	99.53%	690,870	99.26%
chr13	525,417	524,754	99.87%	613	0.13%	50	523,496	99.63%	521,409	99.24%
chr14	472,985	470,625	99.50%	756	0.50%	1,604	470,623	99.50%	469,242	99.21%
chr15	426,670	425,270	99.67%	1,175	0.33%	225	425,201	99.66%	423,901	99.35%
chr16	454,875	450,948	99.14%	3,903	0.86%	24	450,658	99.07%	449,262	98.77%
chr17	394,768	388,705	98.46%	4,633	1.54%	1,430	388,188	98.33%	387,257	98.10%
chr18	415,068	414,880	99.95%	128	0.05%	60	414,857	99.95%	413,357	99.59%
chr19	327,725	325,617	99.36%	692	0.64%	1,416	325,612	99.36%	325,085	99.19%
chr20	324,182	319,625	98.59%	4,350	1.41%	207	319,261	98.48%	318,579	98.27%
chr21	201,577	200,118	99.28%	1,361	0.72%	98	199,148	98.80%	198,695	98.57%
chr22	204,523	198,019	96.82%	5,168	3.18%	1,336	194,093	94.90%	193,774	94.74%
**Total**	**14,513,665**	**14,438,051**	**99.48%**	**59,506**	**0.41%**		**14,426,613**	**99.40%**	**14,386,673**	**99.13%**

Table S1: Variant summary statistics filtering, liftOver and phasing of the TGP-HGDP Joint Dataset from GRCh38 to GRCh37. The original dataset, released in GRCh38, was first filtered to retain only biallelic SNPs with MAF > 0.5%, forming the baseline set reported in the "*Original number of variants*" column. All subsequent percentages are relative to

this set. A total of 99.48% of variants were successfully lifted over to GRCh37, while 0.41% failed to map. To ensure positional accuracy, we retained only variants that mapped to the same chromosome as their original location, yielding 99.40% in the "LiftOver (Post-Filters)" set. This filtered dataset was then phased using SHAPEIT5 (`phase_common`) with a consistent MAF filter, resulting in a final retention of 99.13% of the original variants. This high-quality GRCh37-converted reference set was used for downstream phasing and local ancestry inference.

#	UKBB Phenocode	Phenotype Description	Total Cases
1	30000	White blood cell (leukocyte) count	5564
2	30060	Mean corpuscular haemoglobin concentration	5564
3	30600	Albumin	5418
4	30610	Alkaline phosphatase	5840
5	30620	Alanine aminotransferase	5838
6	30630	Apolipoprotein A	5390
7	30640	Apolipoprotein B	5795
8	30650	Aspartate aminotransferase	5806
9	30660	Direct bilirubin	4865
10	30670	Urea	5837
11	30680	Calcium	5416
12	30690	Cholesterol	5836
13	30700	Creatinine	5836
14	30710	C-reactive protein	5827
15	30720	Cystatin C	5835
16	30730	Gamma glutamyltransferase	5835
17	30740	Glucose	5409
18	30750	Glycated haemoglobin (HbA1c)	4667
19	30760	HDL cholesterol	5412
20	30770	IGF-1	5800
21	30780	LDL direct	5825
22	30790	Lipoprotein A	4843
23	30800	Oestradiol	1514
24	30810	Phosphate	5408
25	30820	Rheumatoid factor	441
26	30830	SHBG	5369
27	30840	Total bilirubin	5802
28	30850	Testosterone	5292
29	30860	Total protein	5417
30	30870	Triglycerides	5835
31	30880	Urate	5831
32	30890	Vitamin D	5680

Table S2: Summary of Phenotypes Analyzed in Tractor GWAS of UK Biobank Admixed AFR-EUR Individuals. List of all 32 blood-biomarkers analyzed using Tractor GWAS in an admixed AFR-EUR cohort from the UK Biobank along with its corresponding UK Biobank phenocode, description of the phenotype, and the number of

individuals with available data used in the analysis

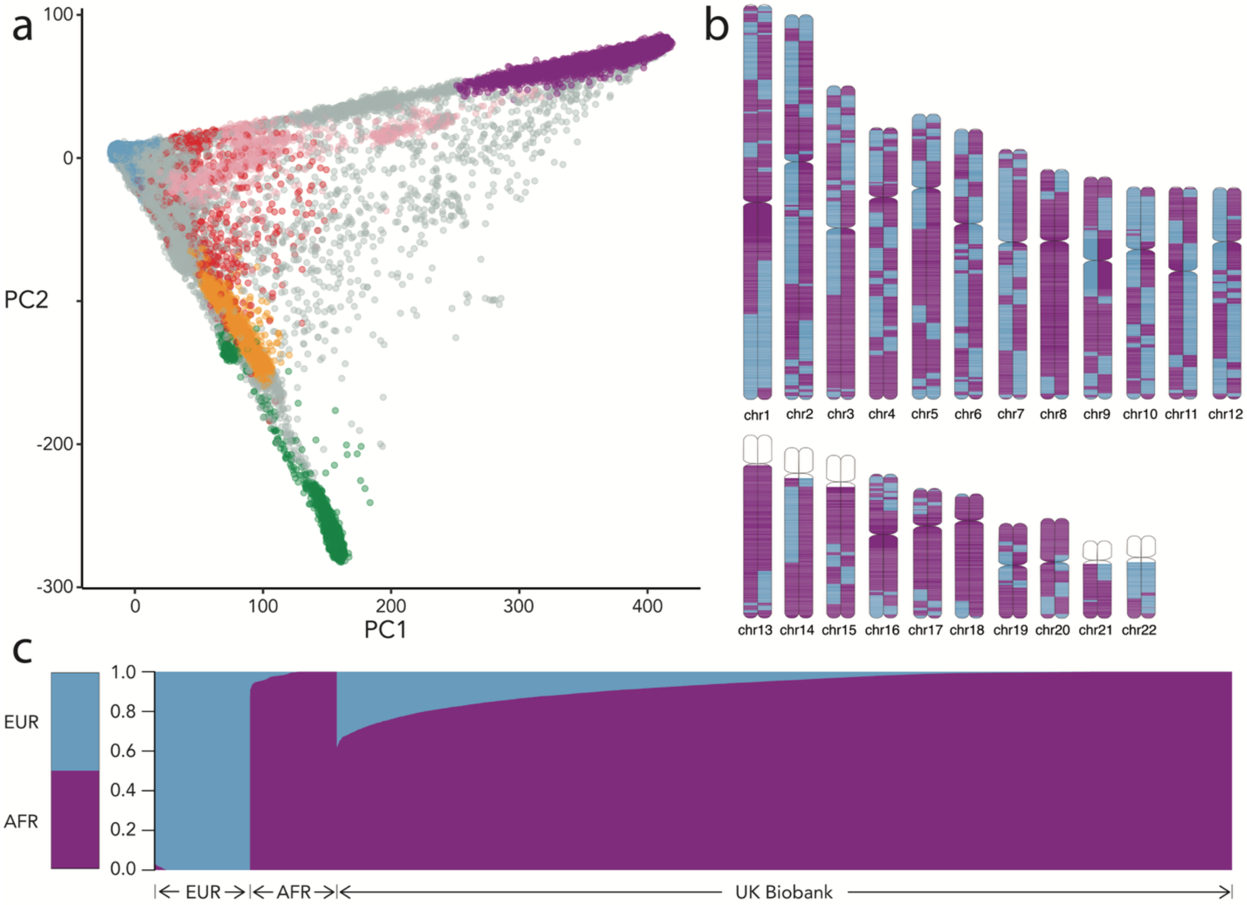

Figure S1: Overview of cohort selection and ancestry inference for Tractor GWAS in UK Biobank. (a) Principal Component (PCA) plot of all UK Biobank individuals. Individuals classified as AFR by Pan-UKBB[17] (purple points) were selected for inclusion in Tractor analyses. (b) Local Ancestry for one of the selected admixed individuals as inferred by RFMix2 showing segmental ancestry along the genome with alternating AFR and EUR tracts (c) ADMIXTURE plot (K=2) showing ancestry proportions for the final set of 6,245 admixed AFR-EUR individuals, alongside AFR and EUR reference samples on the left from the TGP-HGDP joint-call dataset.

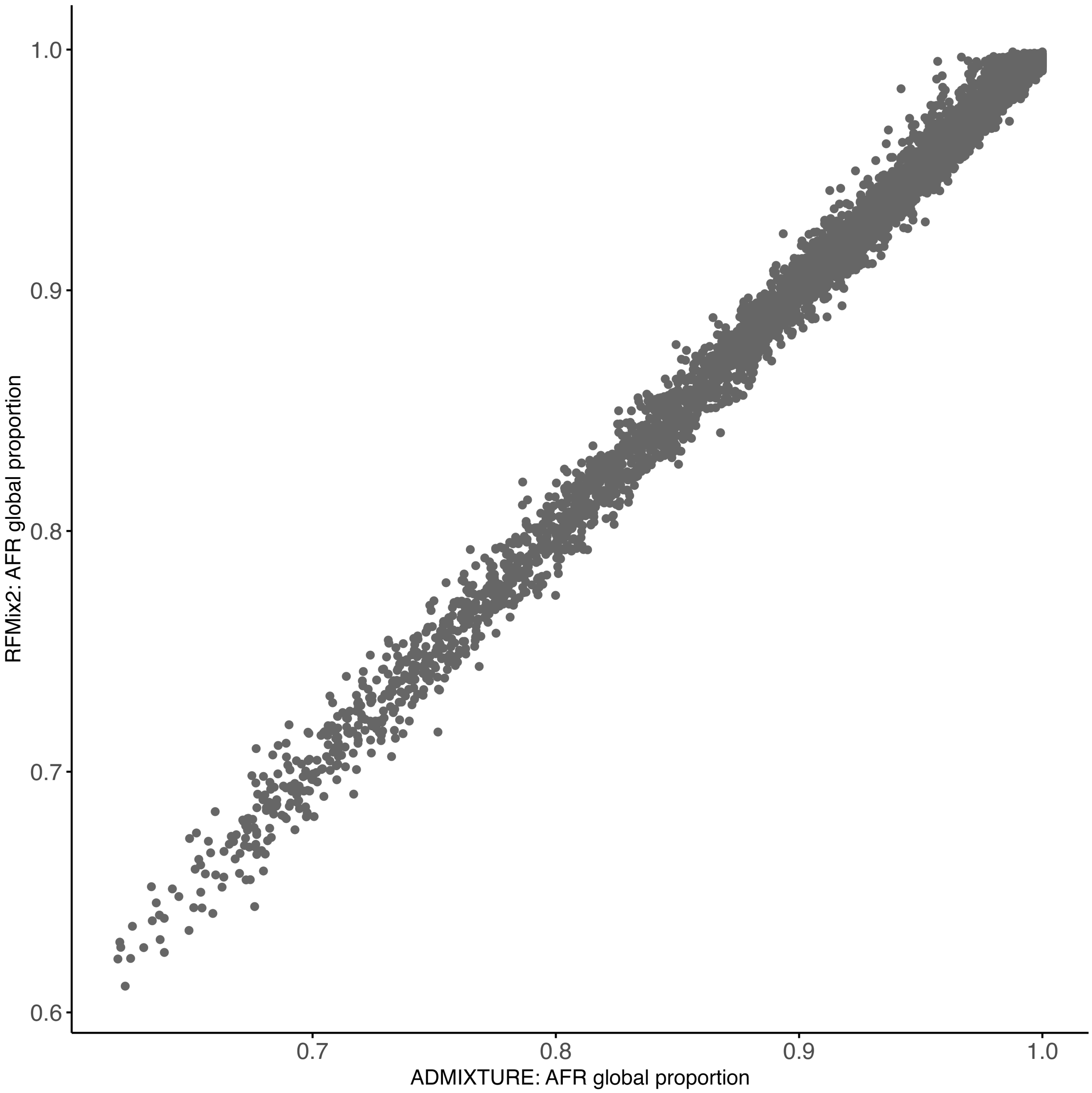

Figure S2: Strong correlation between lobal Ancestry Estimates from ADMIXTURE and RFMix2 Supports Accuracy of Local Ancestry Inference using RFMix2. Global ancestry proportions estimated using ADMIXTURE (K=2) were compared to global ancestry estimates from RFMix2 in 6,245 admixed AFR-EUR individuals from the UK Biobank. The two methods demonstrated strong concordance ($r^2 > 0.99$), supporting the accuracy and reliability of RFMix2's local ancestry inference in this cohort.

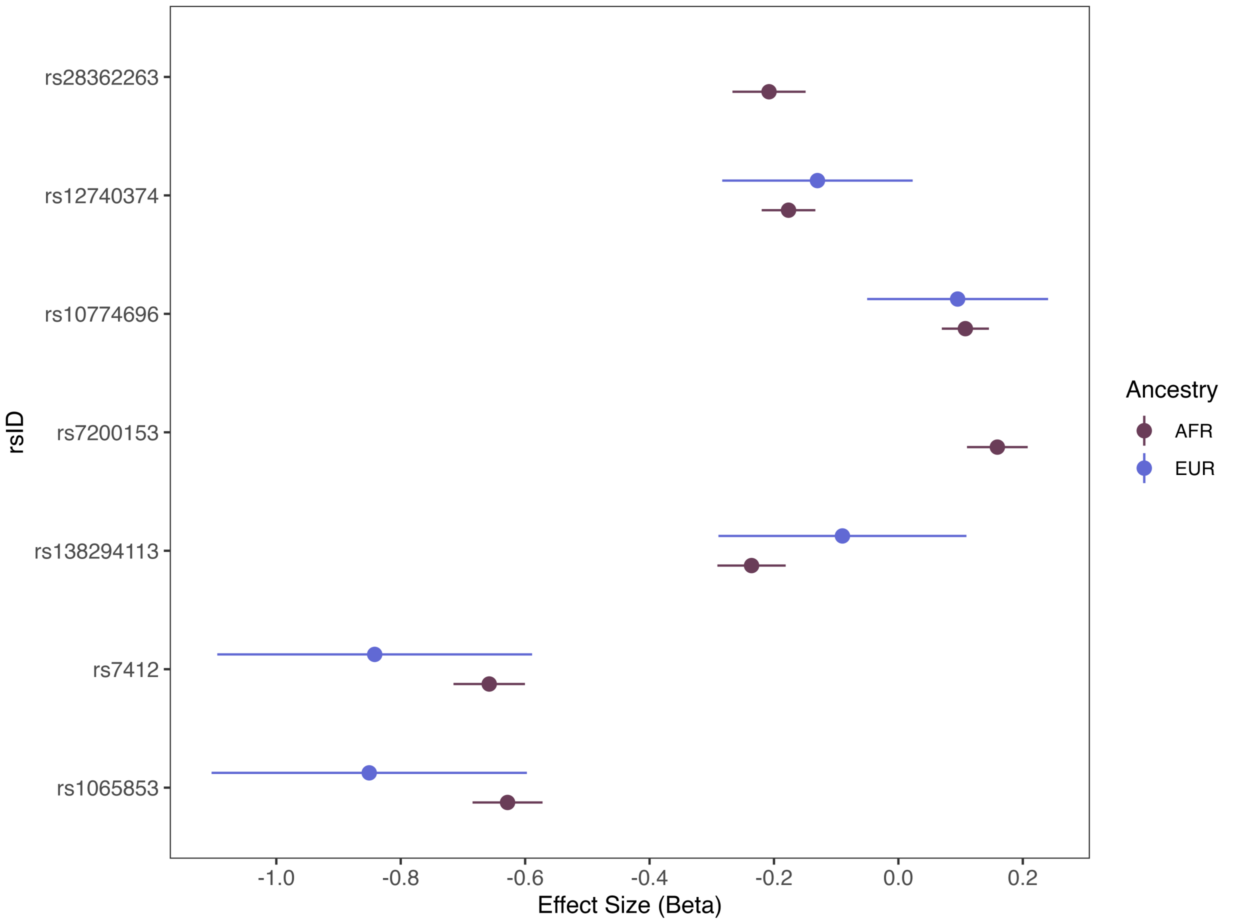

Figure S3: Comparison of ancestry-specific effect sizes estimates for top genome-wide significant loci by ancestry for ApoB trait. Effect size estimates (β) with 95% confidence intervals are shown for each ancestry from the *Tractor* GWAS of ApoB in admixed AFR-EUR individuals from the UK Biobank. Two SNPs (rs28362263 and rs7200153) were observed exclusively in AFR tracts, and thus no corresponding beta estimates were generated for EUR ancestral tracts. Other loci have beta estimates both ancestries, illustrating *Tractor's* ability to detect ancestry-specific associations. These SNPs correspond to those top loci highlighted in Table 2.
